# Monitoring of HIV testing services in the EU/EEA

**DOI:** 10.2807/1560-7917.ES.2016.21.48.30410

**Published:** 2016-12-01

**Authors:** Lara Tavoschi, David Hales

**Affiliations:** 1 European Centre for Disease Prevention and Control (ECDC), Stockholm, Sweden; 2 Independent consultant

**Keywords:** HIV

## Background

HIV continues to be a serious public health issue in the European Union/European Economic Area (EU/EEA) and, despite concerted prevention efforts, the number of new HIV diagnoses reported each year has remained largely unchanged over the last decade [1]. The European region is increasing its efforts to reach the 90–90–90 targets advocated by the Joint United Nations Programme on HIV/AIDS (UNAIDS) [2]. One of the major challenges many European countries face is the high proportion of undiagnosed people living with HIV [3] and the high rates of late diagnosis [4,5]. In the past years, testing programmes have improved in terms of their accessibility and coverage, yet it remains difficult to monitor and evaluate the performance of testing programmes at all levels as a consequence of significant gaps in the data available on testing services [4,6].

In October 2016, the European Centre for Disease Prevention and Control (ECDC) convened an expert consultation, attended by representatives from a range of constituencies (national institutions, community organisations, healthcare workers) from 14 Member States and international organisations, to explore how to strengthen monitoring of HIV testing in the EU/EEA. The consultation’s aims were to (i) share experiences on how HIV testing is currently monitored, (ii) reflect on the need, scope and feasibility of a common approach to monitor HIV testing and (iii) formulate recommendations on how to improve the monitoring of HIV testing in the EU/EEA.

## Strategic information and targeted HIV testing: what is needed?

Representatives of different constituencies from four countries made paired presentations on the need for and the use of strategic information at country level. For each country there were unique positions on the challenges of collecting and using strategic information but a clear consensus emerged that more and better national data were needed to monitor and implement an effective HIV testing strategy.

Susan Cowan (Statens Serum Institut, Denmark) and Per Slaaen Kaye (AIDS-Fondet) emphasised the importance of pushing beyond existing approaches to HIV testing in order to reduce the number of undiagnosed people. They noted that alternative approaches to testing, including, for example, home testing, are likely to be even more difficult to monitor than existing approaches. Cost-per-test and cost-per-case-detected is considered an important element in the assessment of testing approaches. Nevertheless, it was noted that the cost-per-case of finding new cases is likely to increase as the number of undiagnosed people declines.

Florence Lot (Agence nationale de santé publique, France) and Richard Stranz (AIDES) made complementary presentations about the current situation in France. They shared concerns about the large number of undiagnosed HIV cases and the high rates of undiagnosed prevalence among three populations: men who have sex with men (MSM), heterosexual women born abroad and heterosexual men born abroad. Intense community outreach and localised testing are being implemented in France to improve knowledge and testing uptake among these key populations.

Olivia Castillo Soria (Ministry of Health, Social Services and Equality, Spain) and Jordi Casabona (Centre d'Estudis Epidemiològics sobre les ITS i la Sida de Catalunya) described the importance of community HIV testing in Spain and presented an ongoing ministerial initiative to map and geo-reference community-based testing sites in the country and collect standardised information on HIV community testing programmes like number of test and result, testing and counselling and linkage to care. The long-standing experience of the HIV-DEVO Project [7] in Catalonia was presented as an example of a successful approach to monitor community-based testing. According to the latest data ca 20% of the new HIV cases in the region were diagnosed within the network.

Alison Brown (Public Health England), Cary James (Terence Higgins Trust) and Ann Sullivan (Chelsea and Westminster Hospital) presented on the challenges and opportunities for expanded testing in the UK in the context of high rates of undiagnosed and late diagnosis of HIV. New testing guidelines developed by the National Institute for Health and Care Excellence (NICE), to be released in December 2016, recognise the importance of expanding HIV testing outside of traditional settings. Innovative approaches such as home sampling and self-testing have great potential, with one initiative managing to distribute ca 4,000 self-tests in only 10 days in the country. A majority of those using the tests shared their results afterwards, providing a positive indication in terms of monitoring opportunities of this testing approach.

Key points identified through the presentations and ensuing discussion included:

• Taking a pragmatic approach and making use of readily available data, including surveillance and programmatic data are highly important.

• Better estimates of key population size, their geographic distribution within countries, and the relative proportion of undiagnosed cases are crucial to target testing services.

• The substantial contribution of community-based testing in detecting new HIV cases where it has been introduced at scale, e.g. Spain, France, Greece and Portugal, was recognised. It was noted that, while community testing sites often generate good monitoring data, the lack of consistency in the metrics used across single sites undermines the ability to estimate the relative contribution to overall testing efforts in a country, with some notable exceptions at national (e.g. Rede de Rastreio, http://www.gatportugal.org/noticias/rede-de-rastreio-comunitaria-resultados_83, Portugal), sub-national (e.g. the HIV-DEVO Project, Spain) and European level (HIV community-based testing practices in Europe [HIV-COBATEST] network) (8).

## Existing efforts to monitor HIV testing

Representatives from three countries and two EU projects presented their experiences in monitoring HIV testing: Magdalena Pylli (Hellenic Centre for Diseases Control and Prevention, Greece), Kristi Rüütel (National Institute for Health Development, Estonia) and Daniel Simões (Grupo de Ativistas em Tratamentos (GAT), Portugal); Dorthe Raben (Optimising testing and linkage to care for HIV (OptTEST: http://opttest.eu/)) and Laura Fernàndez-López (Operational knowledge to improve HIV early diagnosis and treatment among vulnerable groups in Europe (Euro HIV-EDAT: https://eurohivedat.eu/)). There were substantial similarities in the metrics collected for monitoring HIV testing across the presentations, the most common being the number of screening tests, confirmatory assays, positive tests, reason(s) for testing, sex, age and site/setting (e.g. laboratory, community site, hospital, ante-natal care services). Other metrics were additional socio-demographic data, previous testing history, risk behaviour and risk exposure, knowledge and use of biomedical HIV prevention (e.g. Post-Exposure Prophylaxis), linkage to care, cost per test and cost per diagnosis. Furthermore, measuring the rate at which service providers offer HIV test to eligible patients was deemed a key monitoring element for indicator-condition guided testing(9).

A number of data sources were mentioned, including reference laboratories and primary laboratories, health facilities, hospitals, national health insurance databases, cross-sectional and ad hoc studies, national and international networks of community-based testing sites that collect monitoring data using online platforms [8]. Among the challenges, poor data quality, uncertainty around representativeness of the data, limited availability and implementation of quality control measures were mentioned by all presenters. Lack of integration with national monitoring data was identified as a major challenge for community-based and indicator-condition guided testing initiatives. Additional challenges identified included: (i) tracking retesting by the same individual, (ii) lack of demographic data from anonymous tests, and (iii) the (lack of) reporting culture among service providers and limited financial resources.

## Suitable metrics and data sources for monitoring HIV testing in the EU/EEA

In working groups, participants focused on suitable metrics and data sources for monitoring HIV testing in the EU/EEA.

Key recommendations concerning metrics and data sources included:

• Promote the use of a limited number of metrics that can be easily and widely tracked. There was general consensus around four metrics: (i) number of tests, (ii) basic demographic data of the tester (e.g. age, sex and population group), (iii) location/setting of the test, and (iv) number of reactive/positive tests.

• Use existing data sources to limit additional burden. While the exact data sources will vary by country, existing ones should be able to generate the data for the core metrics.

• Integrate all applicable data in a country to produce meaningful national datasets that capture the activities of the various organisations conducting HIV tests. The critical example is the integration of national monitoring data with those generated from community-based testing sites. It was suggested to promote collation of community-based organisations’ data at country level as a first step towards effective integration with national data.

• Determine how to integrate data on home sampling and self-testing into the monitoring approach. One suggestion was to work with industry/private sector to collect indicative (e.g. sales) data.

The participants agreed that the core metrics should be scalable and flexible. In terms of scalability, the metrics would need to be feasible and meaningful to collect at the site level (e.g. by contributing to quality improvement cycle) but could also be scaled up for use at national and international levels with a comparable level of usefulness. They should also be flexible to allow use in specific settings and with specific populations, such as a network of community-based testing sites serving MSM or of health facilities implementing indicator-condition guided testing which can collect, aggregate and compare data points from these metrics to assess the implementation and effectiveness of the initiatives.

Several other metrics were identified as potentially useful for monitoring HIV testing, including linkage to care, site/setting of first reactive test/diagnosis, and reason for test. Linkage to care was recognised to be a vital data point for community organisations to monitor the ability of effectively referring to care of newly diagnosed individuals. There was general agreement that site/setting of first reactive test and/or diagnosis could be collected as an additional variable within the European HIV surveillance dataset to gather information on testing sites (as a proxy for testing modalities) and their relative yield of positive diagnoses.

While there was consensus that key populations’ size estimates and relative undiagnosed fractions would be extremely valuable instruments to monitor impact of testing programmes, there were concerns about their accuracy and robustness. The ECDC HIV modelling tool [9] [10] [8] is a valuable asset in supporting Members States with a standardised method and an easy-to-use online tool to produce national estimates. Data on late HIV diagnoses and the relative proportion among key populations were considered complementary metrics that could effectively inform targeted interventions.

## Conclusions

Expanding the availability and improving the targeting of HIV testing will reduce the percentage of late HIV diagnoses as well as the overall number of undiagnosed cases in EU/EEA countries. Among the innovative modalities of HIV testing, self-sampling and self-testing programmes as well as community-based voluntary counselling and testing have been shown to expand availability and improve targeting of HIV testing, particularly among key populations who are most affected by HIV.

Improving testing policies, planning, resource allocation and programme performance needs timely, accurate and high-quality data on HIV testing locally, nationally and regionally. Continuous efforts in developing accurate and robust estimates of people living with HIV, the size of key populations and relative undiagnosed fractions should be pursued to enable better assessment of the impact of testing activities. Increasing the utility of already collected metrics, such as the proportion of late diagnoses, could effectively help targeting testing efforts to key sub-groups.

A small, core set of metrics that are straightforward to collect and are broadly useful have been identified and should strengthen the capacity to monitor and evaluate testing programmes at local, national and regional levels. Data from the full range of national HIV testing initiatives, particularly healthcare and community activities, should be routinely aggregated where available to ensure that countries have a complete picture of their situation. The ongoing separation of datasets undermines the ability of all stakeholders to understand and assess the opportunities and challenges facing HIV testing programmes.

## *Erratum

The author name of David Hales was added on 6 December 2016.
