# Erratum for Euro Surveill. 2016;21(48)

**DOI:** 10.2807/1560-7917.ES.2016.21.49.30421

**Published:** 2016-12-08

**Authors:** Eurosurveillance editorial team

**Affiliations:** 1 European Centre for Disease Prevention and Control (ECDC), Stockholm, Sweden

In the article ‘Monitoring of HIV testing services in the EU/EEA' by L Tavoschi et al., published on 1 December 2016, the author name of David Hales was added on 6 December 2016.

